# Group cognitive behavioral therapy for children and adolescents with ADHD

**DOI:** 10.1186/s41155-017-0063-y

**Published:** 2017-05-16

**Authors:** Luzia Flavia Coelho, Deise Lima Fernandes Barbosa, Sueli Rizzutti, Orlando Francisco Amodeo Bueno, Monica Carolina Miranda

**Affiliations:** 1 Psychobiology Department, Universidade Federal de São Paulo, São Paulo, Brazil; 2 Psychology Department, Universidade Federal do Rio Grande do Norte, Natal, Brazil; 3 R Duarte de Azevedo, 448 sala 113, São Paulo, SP CEP 02036-021 Brazil

**Keywords:** Attention-deficit/hyperactivity disorder, Cognitive behavioral therapy, Medication, Treatment, Children

## Abstract

The present study analyzed the use of group CBT protocol to treat ADHD by comparing two types of treatment, unimodal (medication only) and multimodal (medication combined with CBT), in terms of their effects on cognitive and behavioral domains, social skills, and type of treatment effect by ADHD subtype. Participants were 60 children with ADHD, subtypes inattentive and combined, aged 7 to 14, 48 boys. Combined treatment included 20 CBT sessions while all children were given Ritalin LA® 20 mg. Cognitive and behavioral outcome measures showed no differences between treatment groups. On social skills, multimodal showed more improvement in frequency indicators on empathy, assertiveness, and self-control subscales and in the difficulty on assertiveness and self-control subscales. Using a group CBT protocol for multimodal ADHD treatment may improve patient adherence and ADHD peripheral symptoms.

## Background

Attention-deficit/hyperactivity disorder (ADHD) is the most frequent childhood neurobiological disorder with estimated worldwide prevalence at about 3.4% (Polanczyk, Salum, Sugaya, Caye, & Rohde, 2015). The current DSM-5 diagnostic criteria feature three forms of presentation: ADHD/I (predominantly inattentive), ADHD/H (hyperactive and impulsive), and ADHD/C (combined), each with different specific difficulties and responses to treatment (Grizenko, Paci, & Joober, 2010).

ADHD has an unfavorable prognosis if left untreated. Clinical trials conducted since the early 1990s have shown that pharmacological treatment using psychostimulants in particular alleviates ADHD core symptoms and academic and behavioral problems while lowering risk of other ADHD comorbid psychopathologies (MTA Cooperative Group, 1999). However, non-pharmacological interventions combined with pharmacotherapy have alleviated ADHD’s long-term quality-of-life impacts on patients and families (Majewicz-Hefley & Carlson, 2007; Pelham & Gnagy, 1999; Wolraich et al., 2011).

Over the last few years, cognitive behavioral therapy (CBT) has been one of the most extensively researched approaches (Fabiano, 2009; Hodgson, Hutchinson, & Denson, 2014, Majewicz-Hefley & Carlson, 2007; Young, 2013). But there have been few studies of group treatment, which may pose a low-cost alternative to individual therapy in developing countries where access to psychotherapy is scarce due to its high cost (NICE, 2009; Young, 2013).

Group protocols have included the Summer Treatment Program (STP) of eight consecutive weeks of daily treatments using behavioral management practices and social-skill training, which has reported improved academics and peer interventions (Pelham, Greiner, & Gnagy, 1997). A protocol initially developed for adults by Safren et al., (2005) but tested on adolescents (Antshel, Faraone & Gordon et al., 2014) was modeled on motivational interview components covering psychoeducation, organization and planning, distraction, and regulating mood swings (associated anxiety and depression). In addition, the RAPID protocol was developed for schools treating attentional and emotional control skills, problem-solving, and social skills while boosting academic performance (Young, 2013).

Several studies have tested CBT’s efficacy for children with ADHD. The “Multimodal Treatment Study of Children with ADHD” (MTA) (2009) tracked a sample of 579 children to evaluate a 14-month intervention in four treatment groups (medication strategy, behavioral therapy, combination of both treatments, and community care). The medication and combined groups showed significantly more improvement than the others. However, the combined treatment used lower levels of medication than the medication group, while showing more adherence to treatment (MTA Cooperative Group, 1999).

In an alternative analysis of the results from the same MTA sample, Conners et al. (2001) asked whether the outcome variables selected could influence intervention effects. Their factor analysis of key components, followed by a variance analysis comparing the effects of the four types of treatment, showed statistically significant differences between combined and other treatments and the former led to greater short- and long-term benefits. The authors argued that an extremely important aspect when analyzing efficacy of different types of treatment (combined and separate) was the researchers’ choice of outcome measures that may decisively influence results and lead to erroneous interpretations.

The first meta-analysis of behavioral modification treatments, by Fabiano et al. (2009), found effect sizes varying with different study designs. Effect size was greater for the between-group design study (behavioral therapy and control). Evaluations of pre- and post-treatment measures pointed to a moderate effect size, relatively greater in the within-subject and single-subject studies. These authors suggest efforts to disseminate behavioral interventions in community, school, and mental health settings.

In another meta-analysis, Hodgson et al. (2014) evaluated seven types of intervention for children and adolescents with ADHD (behavioral modification, neurofeedback, multimodal psychosocial treatment, school-based programs, memory improvement techniques, self-monitoring, and parental guidance). In terms of statistical significance, a different pattern emerged in which behavioral modification and neurofeedback led to statistically significant improvement. Conversely, a meta-analysis of randomized clinical trials showed the efficacy of non-pharmacological treatments, including dietary and psychological approaches (Sonuga-Barke et al., 2013).

Specifically in relation to behavioral treatment, the authors argue that its effect size is near zero for blind RCTs, unlike other reviews (Fabiano et al., 2009). The authors conclude that their finding may have reflected parents’ responses to questionnaires used to analyze outcomes, in addition to the strict inclusion criteria used for this meta-analysis. They also suggest that treatment measures may not be sufficiently functional and that this type of evaluation should have the outcomes evaluated focus on functional results (Sonuga-Barke et al., 2013).

Therefore, functional measures capable of distinguishing the impact of activities on patients’ daily lives and their autonomy should be used to evaluate the effects of these interventions, as in neuropsychological rehabilitation programs that distinguish functionality and incapacity components and contextual factors as an interactive evolutionary process using the International Classification of Functioning, Incapacity and Health (ICF) (OMS, 2004; Santos, 2005). Particularly because the literature has shown that ADHD associated with a negative impact on quality of life is a major contributor to the disorder’s adverse peripheral outcomes such as poor academics, interpersonal problems, lack of social skills, and delinquency and substance abuse among adolescents and adults (Barkley, 2006; Belcher, 2014; Hodgson et al., 2014; Rohde & Halpern, 2004).

Importantly, Fabiano, Schatz, Aloe, Chacko, and Chronis-Tuscano (2015) noted that many studies use psychosocial nomenclature but refer to different types of intervention ranging from organizational or social skill to neurocognitive training. Aggregating several nomenclatures and choices of outcome measures into a single effect probably alters results for a meta-analysis of intervention-type effect.

In relation to functional outcomes during a group CBT program for ADHD patients, Coelho et al. (2015) reported that the token-economy technique alleviated behavioral problems. Participants presenting the most severe behaviors were selected, and their parents kept journals for 10 weeks to log their frequency, while using reinforcers for appropriate behaviors and modeling for inappropriate behaviors. Of the 11 behavioral categories analyzed, seven showed significant effects in terms of reduced frequency (impulsivity, hyperactivity, disorganization, disobeying rules and routine, poor self-care, easily frustrated, anti-social behavior) in the course of treatment.

Although the American Academy of Child and Adolescent Psychiatry (AACAP, 2007) and the Latin American consensus recommend using psychostimulant associated with behavioral treatment, there are very limited resources available for behavioral treatment, especially in Latin America (Polanczyk et al., 2008). To the best of our knowledge, only one manual (consisting of 12 individual sections) has been published for the Brazilian population, but its efficacy has yet to be tested (Knapp, Rohde, Lyszkowski, & Johannpeter, 2002).

In 2009 therefore, we started an intervention study to examine the effects of individual and combined treatments on children with ADHD (medication, CBT, attention and working memory training) (Miranda et al., 2011). Since existing programs (Pelham et al., 1997; Safren et al., 2005; Young, 2013) could not be used in our local context, we developed a group CBT protocol for ADHD children and adolescents consisting of 20 weeks of treatment based on guidelines from the literature (Barkley, 2006; Mrug et al., 2009; Pelham et al., 1997). The protocol was designed for group use mainly because treating larger number of patients is beneficial for healthcare systems such as those of Brazil and similar countries.

The present study therefore analyzed the group CBT protocol for treating ADHD to compare unimodal (medication strategy) and multimodal (medication combined with CBT) treatments in cognitive (attention and working memory) and behavioral domains (parent and teacher questionnaires) and social skills (child self-reporting), also examining treatment-type effect by ADHD subtype.

## Methods

### Design

This is a non-randomized, parallel, open therapeutic clinical trial with two arms.

### Participants

Children selected were aged 7 to 14, with signs of ADHD as primary disorder and no signs of neurodevelopmental delay (intellectual disability [IQ below 79], epilepsy, genetic syndromes, HIV, hydrocephalus, brain damage, etc.), and not currently taking other medications.

**Fig. 1:**
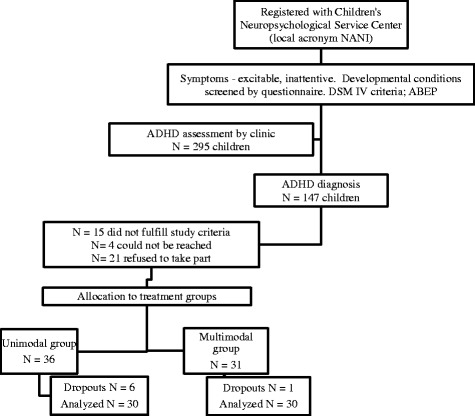
Flowchart. Note: 41 children were directed to other intervention studies (other types) at the center during this period

The children were recruited from a public-system outpatient clinic specialized in diagnosis of children and adolescents with neurodevelopmental disorders associated with Universidade Federal de São Paulo (UNIFESP-SP-Brazil), which specializes in diagnosing children and adolescents with neurodevelopmental disorders. The participants were selected after their parents/guardians spontaneously registered them due to symptoms such as excitability or difficulty keeping quiet and paying attention. A subsequent interview screened for neurodevelopmental aspects, DSM-IV criteria, and socioeconomic status (www.abep.org). Children meeting the initial criteria were submitted to diagnostic assessments and asked to participate as shown in Fig. 1. The neuropsychological evaluation included the following: the children’s intellectual level was tested using the abbreviated (estimated IQ) Wechsler Intelligence Scale for Children (WISC-III), the attention test using the Conners’ Continuous Performance Test (CCPT), the Automated Working Memory Assessment (AWMA) test, and the BRIEF (Behaviour Rating Inventory of Executive Functions) test. The psychiatric interview included a Brazilian version of MTA-SNAP-IV, the Child Behaviour Checklist (CBCL), and the Brazilian version of the Conners Rating Scale (see Rizzutti et al., 2015—for more details)

The participants were pseudo-randomly allocated to treatment groups (unimodal-medication; multimodal-medication combined with cognitive behavioral therapy). Similar numbers of participants diagnosed for each subtype (ADHD/I and ADHD/C) were placed in each treatment group. In addition, groups were organized around family member availability and school schedules. Each treatment group was sampled in the period from 2010 to 2014, due to the fact that the multimodal group treatment was applied to groups of five to six children at most. Both groups were treated from 2011 to 2015.

The final sample analyzed consisted of 60 participants with ADHD, of whom six children dropped out from the unimodal group but only one from the multimodal. The average age was 10.13 (SD 2.11) for the unimodal group and 10.2 (SD 1.86) for the multimodal group, which contained 26 and 22 boys, respectively. In relation to subtypes, 57% of the unimodal group were ADHD/C subtype against 50% of the multimodal group. In terms of socioeconomic status, 48.6% of the unimodal and 40% of the multimodal group belonged to class C. Statistical analysis showed that there were no differences in characterization of the groups (*X* ^2^ = 0.15) or age (*X* ^2^ = 0.82), gender (*X* ^2^ = 0.14), IQ (*X* ^2^ = 0.98), or socioeconomic status (*X* ^2^ = 0.72) (Table 1).

**Table 1:** Sample description

Unimodal group				Multimodal group			
Participants *N* = 30				Participants *N* = 30			
	Mean	SD	%		Mean	SD	%
Age	10.13	2.11		Age	10.2	1.86	
Gender			75 boys	Gender			73 boys
IQ	108.64	15.56		IQ	108.03	13.82	
Subtype			57 (ADHD/C)	Subtype			50 (ADHD/C)
			43 (ADHD/I)				50 (ADHD/I)
Socioeconomic status			5.7 (A1–A2)	Socioeconomic status			20 (A1–A2)
			40 (B1–B2)				30 (B1–B2)
			48.6 (C)				40 (C)
			5.7 (D)				10 (D)

All procedures used were approved by the Ethics Committee of Universidade Federal de São Paulo (ref. CAAE: 00568612.3.0000.5505). Parents/guardians and children signed informed consent forms (UTN: U1111-1145-6707; retrospectively registered 15 July 2013).

### Treatment

#### Medication

**Fig. 2:**
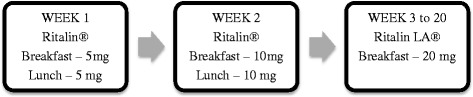
Progressive administration of methylphenidate

Both groups (unimodal and multimodal) were medicated with prolonged-release methylphenidate 20 mg (Ritalin LA®) for 20 weeks. The first fortnight was an adaptation period using immediate release methylphenidate 10 mg (Ritalin®). In week 1, 5-mg doses were administered after breakfast and after lunch each day. In week 2, 10-mg doses were administered after breakfast and after lunch. After the adjustment period, the standardized dose was a single dose after breakfast each day for 18 weeks with methylphenidate extended-release 20 mg (Ritalin LA®) for a total of 20 weeks (Fig. 2).

Once a month after the methylphenidate adaptation period, a doctor checked for any side effects that might impede continued medication and offered advice to alleviate poor appetite, sleep, or other problems in order to better adjust treatment. Medication was provided free of charge.

### Group cognitive behavioral therapy

The CBT protocol developed here based on CBT theoretical principles and existing ADHD programs (Barkley et al., 2008; Berger et al., 2008; Fabiano et al., 2009, DuPaul, Grace & Janusis, 2011; Boo & Prins, 2007; Knapp et al., 2003; Pfiffner, Barkley & DuPaul, 2006; Mocaiber et al., 2008). Six areas were selected as therapeutic goals for the protocol:

- Psychoeducation: ADHD psychoeducation was the subject for the first parent care session (a talk) and the first children’s session (a hyperactive child’s storytelling). There was also psychoeducation based on Beck’s generic cognitive model showing how thought processes influence feelings and behaviors (Beck, 2013).
- Parent training: the main aim for all sessions was advice for family members on establishing routines and healthy habits, using rewards, appreciating behaviors, and handling environments to make them predictable for the children; thoughts, feelings, and cognitive errors related to children; parent behavior and other issues.
- Organizing and planning: parents were shown how to set up a daily routine for a child, schedule commitments (e.g., homework), formulate realistic targets, and split larger tasks into small steps.
- Problem-solving: identifying problems, possible and appropriate solutions to a problem, and consequences of choices.
- Emotional regulation: a few procedures were devised to stimulate the emotional regulation process, supported by CBT techniques such as reinforcement, token economy, self-evaluation, and analyzing thoughts and their relation to behavior.
- Social skills: dramatizing inappropriate situations or behaviors in everyday situations involving peers and teachers, techniques for listening and being heard, and other skills.

For all these therapeutic goals, specific contents were developed (collective rule making, reinforcement framework, teacher communication envelope, self-grading and grading therapists, organizational mural, monthly calendar) as well as techniques such as dramatization, diaphragmatic breathing, and problem-solving.

The protocol proposed initially comprised 28 sessions lasting an hour and a half each, of which 8 were with parents; 20 with children; and 2 with both parents and children. The therapy proposal requires closed groups for five to six children and their families. Three different manuals were compiled to help children, parents, and therapists apply the protocol, then a pilot study tested five ADHD diagnosed children on methylphenidate medication. Eleven protocol sessions were selected to test the structure.

As mentioned above, this protocol was developed in 2009 as part of a larger study (Coelho et al., 2015; Miranda et al., 2011). After its pilot study, there was a need to reduce the number of sessions to ensure this protocol’s structure would match the aims of the larger project. In addition, parents found that handling two manuals (one for parent and another for children) was difficult so we decided to develop a single “patient manual.” The therapist’s manual remained separate but was amended in line with the patient manual, and more descriptions were added to show how sessions should be held. The final CBT protocol consisted of twenty 2-h sessions held weekly. Patient and therapist manuals are being revised for publication.

All sessions followed the same structure traditionally used in this type of psychological therapy (Beck, 2008) and with routines for all meetings. A schedule for each session showed step-by-step sequences of the themes to be addressed. Sessions with families (lasting about 40 min) started with what we called an “impact poster” featuring written sentences related to the issue being discussed (baseless notions concerning medication, behavioral management of children using appropriate reinforcers and thought changers, caregiver behavior). Then, the children started their session (about 80 min) by drawing to show how they were feeling on that day. Next was a review of a suggested homework assignment and tokens (as per the token-economy technique) were introduced in the fifth session. This was followed by a specific activity for the session (problem-solving, self-instruction, planning and organization, perception of feelings and thoughts, perception of consequences, development of socioemotional skills, diaphragm breathing, and relaxation). On concluding the latter, a home activity was suggested. The session ended with self-evaluation (feedback) on behavior during the session scoring from 0 to 10 and the therapist’s evaluation reinforcing the appropriate behavior of each child.

All CBT treatment groups were accompanied by the same specialized psychological professionals (a therapist and a co-therapist). CBT started concurrently with medicamentous treatment.

### Outcome measures

Two different teams conducted pre- and post-treatment evaluations, and the post-treatment evaluation team was blind in relation to children’s characteristics such as diagnosis presented (inattentive or combined), initial results of clinical and neuropsychological evaluation, and which intervention group they had joined. The following measurements were analyzed.

Conners’ Continuous Performance Test (CPT)**—**computerized visual task for evaluating sustained attention (Conners, 2002). The following standardized *T*-score measures were used: omissions, commissions, reaction time standard error, variability, perseverations, reaction time block change, and reaction time inter-stimulus interval change. The measures chosen were based on studies that showed differences in children with ADHD (Miranda et al., 2012).

Automated Working Memory Assessment (AWMA)**—**computerized battery of verbal and visuospatial short-term and working memory tests (Alloway, 2007) using standardized scores for digit recall, listening recall, block recall, spatial recall, and counting span.

Behavior Rating Inventory of Executive Functions (BRIEF; Gioia, Isquith, Guy, & Kenworthy, 2000)—parent and teacher questionnaire for the frequency of behaviors associated with executive function in children’s day-to-day life, version adapted for the Brazilian population (Carim, Miranda, & Bueno, 2012). *T*-scores from the behavioral regulation, metacognition, and global indices were used.

Child Behavior Checklist (CBCL; Achenbach, 1991)—questionnaire assessing social competence and mental health problems in children and adolescents reported by parents/primary caregivers and adapted for the Brazilian population (Bordin et al., 2013). The measures used were internalizing and externalizing problems, total problems, affective problems, anxiety, somatic problems, hyperactivity and inattention, oppositional defiant behavior, and conduct problems.

Teacher-reported Child Behavior Rating Scale (local version acronym EACIP)—scale for five key areas of child behavior (Brito, 2006). Measures standardized by age (*z*-score) were used for hyperactivity/conduct problems, independent functioning, inattention, neuroticism/anxiety, and socialization.

Children’s Social Skills Multimedia System (local version acronym SMHSC-Del-Prette)—behavioral inventory portraying various contexts of everyday school life during interaction with other children and adults using video (Del Prette & Del Prette, 2005). The program produces indicators for frequency, adequacy, and difficulty in relation to the type of reaction: skillful, passive non-skillful, or active non-skillful. These parameters refer to subscales for empathy/civility, assertiveness/coping, self-control, and participation. Each indicator is shown to children in nominal form and then converted to numerical values (0, 1, or 2). The present study analyzed only indicators evaluating skill-related responses from the four subscales. This inventory was introduced in the course of the study due to preliminary results from the larger study (Miranda et al., 2011) and followed suggestions on using functional measures that were found in the literature (Sonuga-Barke et al., 2013).

### Statistical analysis

Firstly, the 33 outcomes’ baseline assessments were compared via paired *t* test (since Kolmogorov–Smirnov testing confirmed normality) for any group baseline difference. The same procedure was applied to other likely covariates such as IQ, socioeconomic status (SES), and age. Chi-square testing was applied to gender and ADHD subtype proportion differences.

Effects of both intervention and ADHD subtypes (dichotomous fixed factors) on post-intervention measurements were assessed via six different GLMs, one for each domain studied: (CBCL [nine dependent variables], CPTT [eight dependent variables], EACIP [five dependent variables], AWMA [five dependent variables], BRIEF parent’ reports [three dependent variables], and teacher reports [three dependent variables]). We did not insert baseline assessments or the abovementioned covariates in the same multivariate model to avoid overfitting (Hawkins, 2004; Zhang, 2014). For example, a regression with at least nine dependent variables (post-intervention outcomes), nine covariates (base outcomes measurements), and two fixed factors would fit CBCL’s GLM. The interaction effect between two fixed factors (moderating effect) was assessed too.

All GLM analyses were performed using SPSS version 22 with 0.05 significance level (*α*). However, if a main effect was found to be statistically significant (using Pillai’s trace), dependent variables showing significant difference across groups were checked if their *p* values were less than *α*/number of dependent variables, as per the procedure recommended by Raykov and Marcoulides (2012). Although this correction might be seen as too conservative, it is recommended when fewer than 10 dependent variables are being tested (Johnson & Wichern, 1992).

As per the CONSORT statement (Schulz, Altman, Moher, & Group, 2010), the present study was not a randomized clinical trial since patients were not allocated to the two arms by a random unpredictable process. No method (true or pseudo) was used to generate a random allocation sequence; children were placed in one group or another for logistical reasons. Due to the non-random allocation to unimodal or multimodal intervention and the biased consequences of a non-random clinical trial (Schulz, Chalmers, Hayes, & Altman, 1995), we adopted a more robust procedure involving estimation of *treatment effect* which is commonly used for quasi-experimental/observational studies that cannot be randomized (for more details, see (Abadie, Drukker, Herr, & Imbens, 2004; Becker & Ichino, 2002). We opted to use inverse probability weighted regression adjustment estimator (Austin, 2011) for more robust findings. Through the treatment effect paradigm, 33 regressions were assessed using STATA version 14.1 (one for each outcome individually), with the level of significance of 0.0015 corrected to avoid false positives as suggested by Wasserstein and Lazar (2016). For inverse probability weighted regression adjustment estimator, the following variables were assumed to predict group allocation: gender, age, ADHD subtype, and IQ. The dependent variables were the post-intervention assessments and their respective baseline assessments.

To analyze social skill outcome measures, variable distribution was deduced by the delta method followed by gamma-distributed linear generalized mixed models (GLMMs) due to the low variance of results emitted by the dependent variable (distribution 0, 1, 2) (Field, 2009). The treatment group was assumed as fixed factor and multimodal treatment as reference group. This analysis used SPSS version 22 with a 0.05 significance level.

## Results

Table 2 shows lack of evidence of mean differences between groups at baseline measurements on *T*-scores for all 33 outcomes. IQ, socioeconomic status (SES), gender frequency, and age were also not statistically significant between groups. Therefore, rather than incorporate baseline outcome measurements and the abovementioned covariates to the model, the analysis focused on key hypothesis testing for intervention effect, subtype effect, and interaction between both fixed factors across the six domains.

**Table 2:** Mean of cognitive and behavior scores pre- and post-intervention by treatment group

	Unimodal				Multimodal			
	(*n* = 30)				(*n* = 30)			
	Pre		Post		Pre		Post	
Outcomes	Mean	SD	Mean	SD	Mean	SD	Mean	SD
CBCL internalizing problems	68.13	6.62	62.40	13.08	65.62	9.42	60.9	9.6
CBCL externalizing problems	68.10	8.83	62.23	13.57	66.14	10.80	60.6	12.3
CBCL total problems	70.97	5.74	65.07	9.82	69.97	8.70	64.1	8.7
CBCL affective problems	69.20	6.93	62.87	13.76	68.14	8.36	64.2	7.8
CBCL anxiety problems	65.70	7.53	61.33	13.29	64.55	8.80	61.4	7.2
CBCL somatic problems	60.80	8.95	56.23	13.15	61.21	10.88	58.6	8.2
CBCL attention/hyperactivity problems	69.93	7.97	64.27	13.62	70.24	8.10	64.5	8.8
CBCL oppositional defiant problems	66.87	8.79	59.70	13.63	64.03	9.90	61.2	9.4
CBCL conduct problems	63.83	14.61	59.73	14.09	65.28	9.57	61.3	8.2
CPT omissions	65.23	21.95	50.90	9.98	64.64	17.38	49.3	11.0
CPT commissions	53.28	9.13	45.83	10.67	54.12	8.15	48.0	11.1
CPT hit reaction time standard error	62.29	12.75	50.06	12.65	63.87	8.83	52.7	9.0
CPT variability	60.27	11.38	48.41	12.83	61.10	6.80	51.4	9.1
CPT detectability	56.34	8.72	47.66	10.39	56.74	7.83	48.9	13.2
CPT perseverations	58.70	16.96	50.49	11.98	68.28	24.44	53.3	15.5
CPT hit reaction time block change	52.46	18.53	50.42	9.17	55.33	13.34	49.9	8.6
CPT reaction time inter-stimulus interval	62.58	15.53	51.48	13.16	60.42	15.14	49.1	7.3
EACIP hyperactivity	1.05	1.27	0.54	1.15	1.30	1.31	1.0	1.1
EACIP independent operation	−0.47	0.93	−0.59	0.89	−0.72	0.85	−0.5	0.9
EACIP inattention	1.64	0.85	0.92	1.06	1.50	1.04	1.0	1.0
EACIP neuroticism/anxiety	0.13	0.98	0.28	1.15	0.30	1.19	0.5	1.2
EACIP socialization	0.05	0.98	0.21	1.17	0.27	1.35	0.3	1.2
AWMA digit recall	90.02	20.74	93.31	15.79	86.34	14.72	83.4	18.3
AWMA listening recall	90.54	15.47	98.23	14.48	85.89	17.89	92.9	13.8
AWMA counting recall	92.62	19.45	100.54	17.22	86.96	14.46	90.7	13.3
AWMA back digit recall	78.13	16.20	80.25	14.73	75.95	13.77	77.5	11.8
AWMA spatial recall	92.24	14.94	101.53	16.06	89.76	14.56	92.8	16.8
BRIEF parent behavioral regulation	65.30	11.66	58.32	12.03	66.93	13.05	60.4	11.8
BRIEF parent metacognition	70.17	9.18	62.04	10.68	71.38	9.44	63.1	8.7
BRIEF parent global executive composite	69.80	9.40	61.43	10.53	73.90	19.98	63.0	9.5
BRIEF teacher behavioral regulation	67.72	14.39	64.22	19.97	75.48	17.80	70.7	16.4
BRIEF teacher metacognition	68.86	23.83	61.00	26.74	61.24	29.67	68.5	12.6
BRIEF teacher global executive composite	68.55	23.68	61.50	26.73	62.90	31.11	70.8	12.7

Box’s test rejected the hypothesis regarding equal covariance matrix; therefore, we decided to report the Pillai’s trace severe distortion in the alpha levels of the tests for the six GLMs.

As Table 3 shows, we have evidence of intervention-group effect for GLM in the AWMA domain (*F*(5.47) = 2.554, *p* value = 0.04; Pillai’s trace = 0.214; partial *η* ^2^ = 0.214). For other domains evaluated, we have lack of evidences for group effect, subtype effect, and interaction between both fixed factors. Due to this group effect on AWMA domain, a between-subject test was conducted across the five dependent variables tested to locate significant effect and find whether the effect is still genuine after the Bonferroni correction (*α*/5 = 0.01; meaning that the level of significance will be 0.01).

**Table 3:** Intervention-group effect and fixed factor interactions

Model	Domains	Group (Pillai’s trace value, *F*)	Group (Pillai’s trace *p* value)	Subtype (Pillai’s trace value)	Subtype (Pillai’s trace *p* value)	Interaction (Pillai’s trace value)	Interaction (Pillai’s trace value)
Main effect	CBCL	1.184, *F* = 1.184	0.326	0.251, *F* = 1.822	0.088		
Main effect	CPT	0.160, *F* = 1.188	0.325	0.075, *F* = 0.505	0.847		
Main effect	EACIP	0.094, *F* = 1.04	0.404	0.154, *F* = 1.826	0.125		
Main effect	AWMA	0.214, *F* = 2.554	0.040	0.080, *F* = 0.813	0.547		
Main effect	BRIEF (parents)	0.008, *F* = 0.143	0.934	0.116, *F* = 2.315	0.086		
Main effect	BRIEF (teacher)	0.080, *F* = 1.476	0.232	0.072, *F* = 1.313	0.280		
Interaction model	CBCL	0.178, *F* = 1.153	0.346	0.251, *F* = 1.785	0.096	0.046, *F* = 0.254	0.984
Interaction model	CPT	0.160, *F* = 1.170	0.336	0.075, *F* = 0.496	0.853	0.092, *F* = 0.619	0.758
Interaction model	EACIP	0.097, *F* = 1.047	0.401	0.157, *F* = 1.819	0.126	0.008, *F* = 0.081	0.995
Interaction model	AWMA	0.219, *F* = 2.587	0.038	0.073, *F* = 0.723	0.610	0.035, *F* = 0.339	0.887
Interaction model	BRIEF (parents)	0.008, *F* = 0.41	0.935	0.124, *F* = 2.443	0.074	0.044, *F* = 0.796	0.502
Interaction model	BRIEF (teacher)	0.084, *F* = 1.535	0.217	0.085, *F* = 1.535	0.213	0.067, *F* = 1.197	0.320

As Table 3 shows, all *p* values were greater than 0.01, so there was no evidence of intervention-group effects in this domain.

Lastly, Table 4 shows the values for the treatment effect using inverse probability weighted regression adjustment; the coefficients obtained express the mean difference between modal versus unimodal intervention. Due to corrected *p* value, no comparison was statistically significant as previously obtained with GLM models. There was no evidence for the comparison between unimodal and multimodal approach. An important point to note is that absence of evidence is not the same as evidence of absence. An a posteriori sample size was calculated for the following input parameters: power (1 − *β*) = 0.8, significance level 0.00015 (regarding the 33 outcomes being tested) for the same sampling.

**Table 4:** Treatment effect using inverse probability weighted regression adjustment

Outcomes	Coefficient (unimodal-multimodal)	Robust standard error	*p* value
CBCL internalizing problems	−0.35	2.39	0.883
CBCL externalizing problems	0.83	3.2	0.795
CBCL total problems	0.40	2.00	0.841
CBCL affective problems	−2.36	2.4	0.326
CBCL anxiety problems	−0.36	2.54	0.887
CBCL somatic problems	−2.29	2.64	0.386
CBCL attention/hyperactivity problems	0.34	2.34	0.882
CBCL oppositional defiant problems	−3.21	2.80	0.252
CBCL conduct problems	−0.86	2.72	0.751
CPT omissions	1.33	2.42	0.583
CPT commissions	−1.69	1.85	0.361
CPT hit reaction time standard error	−2.17	2.38	0.363
CPT variability	−3.12	2.57	0.224
CPT detectability	−1.10	2.80	0.666
CPT perseverations	−0.07	3.15	0.981
CPT hit reaction time block change	0.56	2.35	0.809
CPT reaction time inter-stimulus interval	2.34	2.74	0.397
EACIP hyperactivity	−0.29	0.22	0.188
EACIP independent operation	−0.25	0.18	0.179
EACIP inattention	−0.12	0.24	0.618
EACIP neuroticism/anxiety	−0.14	0.27	0.603
EACIP socialization	0.16	0.24	0.483
AWMA digit recall	8.85	4.09	0.031
AWMA listening recall	4.02	3.39	0.235
AWMA counting recall	7.34	3.67	0.046
AWMA back digit recall	−0.38	2.44	0.875
AWMA spatial recall	7.53	3.88	0.052
BRIEF parent behavioral regulation	−0.36	2.2	0.868
BRIEF parent metacognition	−0.61	2.06	0.767
BRIEF parent global executive composite	0.19	2.47	0.936
BRIEF teacher behavioral regulation	−3.27	3.55	0.357
BRIEF teacher metacognition	−8.41	5.29	0.112
BRIEF teacher global executive composite	10.63	5.34	0.047

Table 5 shows the mean and standard deviations on social-skill test variables for the group pre- and post-treatment. This measure was inserted in the course of the study, thus analyzing 17 unimodal and 15 multimodal group participants.

**Table 5:** Mean pre- and post-treatment social skill scores

Indicators	Subscales	Unimodal				Multimodal			
		(*n* = 17)				(*n* = 15)			
		Pre		Post		Pre		Post	
		Mean	SD	Mean	SD	Mean	SD	Mean	SD
Frequency	Empathy/civility	1.57	0.27	1.61	0.31	1.55	0.35	1.69	0.35
	Assertiveness/coping	1.27	0.29	1.19	0.38	0.88	0.47	1.29	0.54
	Self-control	1.22	0.42	1.32	0.39	1.09	0.42	1.48	0.54
	Participation	1.53	0.37	1.44	0.39	1.29	0.36	1.4	0.49
Adequation	Empathy/civility	1.75	0.5	1.92	0.15	1.88	0.13	1.85	0.31
	Assertiveness/coping	1.54	0.34	1.47	0.36	1.34	0.38	1.44	0.53
	Self-control	1.47	0.29	1.59	0.25	1.53	0.34	1.58	0.44
	Participation	1.87	0.27	1.9	0.15	1.88	0.17	1.78	0.35
Difficulty	Empathy/civility	0.39	0.5	0.44	0.59	0.32	0.37	0.07	0.1
	Assertiveness/coping	0.53	0.47	0.52	0.56	0.29	0.36	0.21	0.3
	Self-control	0.44	0.46	0.49	0.45	0.36	0.38	0.15	0.3
	Participation	0.51	0.49	0.59	0.62	0.42	0.43	0.17	0.29

The analysis showed a significant effect of type of treatment on the empathy/civility subscale frequency indicator (*B* = 0.96, SD = 0.22, *p* = 0.001), the assertiveness/coping subscale (*B* = −0.50, SD = 0.30; *p* = 0.05), and self subscale (*B* = −1.12, SD = 0.12; *p* = 0.001) and that the multimodal group performed better after treatment than the unimodal group.

There was a “type of treatment” effect on the indicator of difficulty on the assertiveness/coping (*B* = 0.96, SD = 0.15, *p* = 0.001) and self-control (*B* = −0.26, SD = 0.11, *p* = 0.020) subscales, showing that the multimodal group had less difficulty after treatment. There was no statistical difference in relation to the adequacy indicator on any of the subscales. (Table 6 shows the detailed description of the statistical model.)

**Table 6:** GLZMM model with Gamma distribution with social skills as a function of group treatment (multimodal group)

Indicators	Subscales	*B*	SD	Wald	*p* value
				Chi-square	
Frequency	Empathy/civility	−0.96	0.22	18.77	0.01
	Assertiveness/coping	−0.9	0.3	12.79	0.05
	Self-control	−1.12	0.12	82.14	0
	Participation	0.17	0.1	2.72	0.01
Adequation	Empathy/civility	−0.1	0.21	0.25	0.62
	Assertiveness/coping	0.17	0.28	0.39	0.53
	Self-control	0.06	0.11	0.36	0.55
	Participation	0.15	0.19	0.63	0.43
Difficulty	Empathy/Civility	0.11	0.19	0.36	0.55
	Assertiveness/Coping	0.29	0.15	3.64	0.05
	Self-control	−0.26	0.11	5.9	0.02
	Participation	−0.02	0.11	0.05	0.83
QICC = 1859.40					

*QICC* corrected quasi likelihood under independence model criterion, *B* betas non-adjusted (time effect), *SD* standard deviation

## Discussion

The purposes of this study were to analyze the use of the group CBT protocol in treatment of ADHD, comparing unimodal (medication only) and multimodal (combined medication and CBT) treatments on cognitive and behavioral domains and social skills and to ascertain the effect of ADHD subtype in response to types of treatment.

The comparison between treatment groups’ pre-intervention showed no differences in standardized cognitive measures (attention and working memory) or behavioral measures. On analyzing the effect of type of treatment between the unimodal and multimodal groups, no evidence was found for outcome measures evaluated in this study, nor were there differences between the ADHD subtypes analyzed. The findings of this study should be analyzed in the light of contradictory findings in the literature on psychosocial treatments. Some studies found no significant effects in multimodal treatments (MTA Cooperative Group, 1999; Sonuga-Barke et al., 2013), whereas others did (Conners et al., 2001; Fabiano et al., 2009).

The MTA group’s study (MTA Cooperative Group, 1999) analyzed results after 14 months of intervention in children with combined subtype ADHD; comparisons across different treatment groups showed that combined treatment did not differ from medicamentous treatment for the six domains analyzed, as in the present study.

Comparing unimodal and multimodal treatment again failed to provide evidence in a meta-analysis conducted only with randomized blind clinical trials which found a size effect of multimodal treatment close to zero (Sonuga-Barke et al., 2013). Similarly, Hodgson et al. (2014) found no effect of behavioral treatment on working memory capacity compared to a control group.

However, there are cases in the literature of significant effects when comparing unimodal and multimodal treatment. Conners et al. (2001) conducted a priori factor analysis; unlike the MTA study, they compared different treatments using combined treatment as reference group which resulted in a smaller effect size than the medication group, moderate compared with behavioral therapy, and large compared with a community care group. The authors suggest that there is a tradeoff when using a composite score, possibly because it is more sensitive to effects on peripheral ADHD symptoms such as social skills and comorbid symptoms.

In terms of the peripheral symptoms of ADHD, this study observed an effect of the multimodal group on measures of social skills. There were higher frequency indicators for skillful reactions on empathy, assertiveness, and self-control subscales, in which the multimodal group showed improvement after treatment as well as reduced perception of difficulties in socially skillful reactions on the assertiveness-difficulty and self-control-difficulty subscales.

Although social-skill scales differ between studies, results may be compared. Conners et al. (2001) found significant results for the factor that includes social skills. Hodgson et al. (2014) also found that their behavioral therapy group did better in terms of sociability than their control group with a lower level of errors on the Matching Familiar Figures Test for cognitive style and reflection impulsivity. In this respect, in relation to assertiveness and self-control frequency indicators used in the present study, cognitive behavioral therapy combined with medication may also ameliorate ADHD’s peripheral symptoms. Additionally, multimodal treatment may offer other benefits such as higher levels of adherence. In this study, there was one dropout in the multimodal group but six in the unimodal, which may show a positive effect of this technique since dropout rates for this population are quite high (MTA Cooperative Group, 1999). Other studies that compared unimodal and multimodal interventions have reported similar findings (Antshel et al. 2014; MTA Cooperative Group, 1999).

These results show the importance of outcome measures in determining treatment effect (Conners et al., 2001). Most studies use standardized measures based on core symptoms of the disorder (ADHD) taken from the International Classification of Diseases (ICD-10) on scales rating symptoms (CBCL, SNAP IV, BRIEF). Presentations of the questions use an affirmative sentence and rate the frequency of behavior shown in the evaluation period. In clinical practice, these scales are used to track psychopathological symptoms with an etiologically based structure with diagnosis by tracking core ADHD symptoms. Therefore, these instruments were not initially developed to evaluate the impact of treatment over time, much less to detect the disorder’s peripheral symptoms.

Therefore, the use of functional measures, as the literature suggests, may provide a clearer view of the effects of cognitive behavioral interventions on ADHD (Coelho et al., 2015; Conners et al., 2001; Sonuga-Barke et al., 2013). A model capable of combining ICF and ICD concepts may shed light on ADHD’s impacts on aspects of health and assess an individual’s different levels in terms of functionality, activities, participation, and their limitations, as well as how environmental factors interact with these constructs. An understanding of how individuals interact with their settings—and how the latter react to their responses—may help to plan treatment and make decisions by broadening and deepening our view of how non-pharmacological interventions affect ADHD core and peripheral symptoms.

Another relevant factor is that future meta-analyses must distinguish nomenclature for these interventions if their effects are to be compared. Since behavioral and cognitive behavioral therapies are based on different conceptual principles, our results cannot be compared to those of other studies to assess the effects of these intervention techniques. Furthermore, having several different outcome measures aggregated in a single effect size may lead to erroneous conclusions as to the efficacy of techniques used to treat ADHD.

From this point of view, functional evaluations of the effects of non-pharmacological intervention may be most suitable for these studies and provide more reliable indicators of the impact of this type of treatment (Coelho et al., 2015; Pelham et al., 2000; Sonuga-Barke et al., 2013; Young, 2013).

## Conclusions

This study showed that the group CBT protocol for ADHD may benefit patient adherence to treatment. Improvements were found in peripheral symptoms of ADHD in the multimodal group and in social skills with increasing frequency on empathy, assertiveness, and self-control subscales and diminished perception of difficulties on the assertiveness and difficulty of self-control subscales.

The findings lacked evidence for treatment group effect when using cognitive (working memory and attention) and behavioral measures. These measures did not show statistical significance and therefore did not evince any clinical or practical significance. However, the absence of significance does not show that the treatments are equivalent; therefore, this should not be seen as a limitation of the study.

Nevertheless, there are other limitations. Generalizing from the present study is difficult due to the small sample drawn from a single center in the city of São Paulo. Another important limitation was the small sample’s narrow age range.

Further research is needed to test this program by evaluating the effect of intervention with a larger sample using functional measures, thus assessing the impact of treatment on daily life for children and families in relation to type of treatment and the generalizing of skills acquired from the program. Other important aspects for future research with this protocol would be to select a larger sample and control results for socioeconomic characteristics (family income, parents’ educational level, and type of school attended by children) and participants’ comorbidities, prior exposure to medication and parental psychopathology. Again, future intervention trials could consider holding simultaneous individual sessions to test effects of social skills.
